# “Metabolic fingerprints” of cachexia in lung cancer patients

**DOI:** 10.1007/s00259-024-06689-8

**Published:** 2024-03-20

**Authors:** Armin Frille, Jann Arends, Elisabetta M. Abenavoli, Shaul A. Duke, Daria Ferrara, Stefan Gruenert, Marcus Hacker, Swen Hesse, Lukas Hofmann, Sune H. Holm, Thomas B. Lund, Michael Rullmann, Peter Sandøe, Roberto Sciagrà, Lalith Kumar Shiyam Sundar, Anke Tönjes, Hubert Wirtz, Josef Yu, Osama Sabri, Thomas Beyer, (the LuCaPET consortium)

**Affiliations:** 1 Department of Respiratory Medicine, Leipzig University, Liebigstrasse 20, 04103 Leipzig, Germany; 2 Department of Medicine I, Faculty of Medicine, Medical Center - University of Freiburg, University of Freiburg, Hugstetter Strasse 55, 79106 Freiburg, Germany; 3 Nuclear Medicine, Azienda Ospedaliero Universitaria Careggi, Largo Brambilla, 3, 50134 Florence, Italy; 4 Department of Food and Resource Economics (IFRO), University of Copenhagen, Rolighedsvej 23, 1958 Frederiksberg C, Copenhagen Denmark; 5 Quantitative Imaging and Medical Physics (QIMP) Team, Medical University of Vienna, Währinger Gürtel 18-20, 1090 Vienna, Austria; 6 Division of Nuclear Medicine, Medical University of Vienna, Währinger Gürtel 18-20, 1090 Vienna, Austria; 7 Department of Nuclear Medicine, Leipzig University, Liebigstrasse 18, 04103 Leipzig, Germany; 8 Department of Veterinary and Animal Sciences, Grønnegårdsvej 8, 1870 Frederiksberg C, Copenhagen Denmark; 9 Department of Endocrine Medicine, Leipzig University, Liebigstrasse 20, 04103 Leipzig, Germany

Dear Sir,

Cachexia with its syndromic signs and symptoms is known to seriously affect the quality of life of cancer patients. Cachexia in cancer patients can halt the continuation of anti-cancer treatments, and thus reduce overall survival [1]. It is estimated that up to 20% of cancer patients die from consequences of malnutrition rather than from the tumor itself [2]. In lung cancer—the leading cause of cancer death worldwide [3]—we observe between 30 and 50% of patients at the time of cancer diagnosis to already fulfill the diagnostic criteria of disease-related malnutrition or cancer cachexia, according to recent consensus guidelines [1, 2, 4].

Being unfit for primary anti-cancer treatment delivers an enormous psychological burden and fateful consequences to these patients. Current clinical guidelines [1, 2, 4, 5] clearly recommend a multimodal approach for the “*identification, prevention, and treatment of reversible elements*”. However, to date, few clinical trials include combinations of separate treatment approaches. Moreover, in clinical practice, cachexia is frequently overlooked, and its prevalence and relevance are severely underestimated [6, 7]. Therefore, efficient treatment concepts against the onset and progression of cachexia are sorely needed but have yet to be established [1, 2]. Moreover, the currently perceived lack of success in treating cachexia may also be a reason for bespoke low clinical awareness.

But what if the current clinical understanding of cachexia does not reflect the true complexity of the underlying pathophysiology and therefore renders existing diagnostic approaches in clinical routine ineffective?

Cachexia in cancer patients has been described as a syndrome a long time ago [8]. While separate definitions of cachexia have been introduced for various patient categories and different chronic diseases [4, 9], they do overlap with definitions of malnutrition and sarcopenia [10–12]; recently, the disease-related form of malnutrition with chronic inflammation has been put on a level with cachexia in cancer patients [13]. In the current clinical routine, this precludes separating cachexia due to cancer from malnutrition due to other causes [10]. This clinical challenge seeds our project, which aims to detect cachexia-specific metabolic patterns to support differentiation from other forms of malnutrition.

Our European consortium was awarded research funding from the *EraPerMED consortium* [14] (LuCaPET project, grant number ERAPerMed_324, “*Clinical decision support for predicting cachexia in cancer patients using hybrid PET/CT imaging*”) to study the effectiveness of ^18^F-fluorodeoxyglucose (FDG) imaging in combination with advanced artificial intelligence (AI) algorithms to study the evolution of inter-organ metabolic relations and from this to predict the onset of cancer-induced cachexia in treatment-naive lung cancer patients [15, 16]. Within the LuCaPET project, we aim to derive organ-specific “*metabolic fingerprints*” from whole-body positron emission tomography/computed tomography (WB-PET/CT) images of lung cancer patients who are at risk of developing cachexia.

Our project comprises the collection and analysis of retrospective and prospective data of lung cancer patients from a European consortium of three clinical imaging sites. A key methodological component is the fully automated image analysis of WB-PET/CT data [17] to derive interorgan networks of lung cancer patients with and without cachexia [18, 19]. These interorgan relations may reveal novel and relevant metabolic patterns and possibly checkpoints that might become predictive in the diagnosis and treatment of cachexia in these patients.

AI systems have been shown to produce accurate and reliable diagnoses, prognoses, and treatment suggestions in medicine [20–23]. However, the opacity of most AI systems challenges trust in clinical decision-making, in particular, when clinicians are unable to explain predictions for individual patients due to AI-black boxing. Furthermore, when AI-generated predictions—such as that of cachexia—cannot be underwritten by normal clinical experience, it may challenge the usual patient–physician relationship. Therefore, our consortium includes experts in AI and ethics who will evaluate conditions that may justify the use of opaque AI output in medical decision-making [24]. We argue that within an evidence-based medicine framework, clinicians must have access to explanations of predictions to meet ethical commitments of shared decision-making. Therefore, an additional goal of our project is to examine sociologically how to make AI-driven clinical decision support models detect cachexia transparent and comprehensible for clinicians.

In conclusion, the overarching vision of the *LuCaPET consortium* is to describe lung cancer-induced cachexia as a targetable and treatable condition at the same time as lung cancer patients receive their diagnosis. Toward this goal, we are employing FDG-enhanced WB-PET/CT imaging and a fully automated organ segmentation analysis. We are currently testing whether resulting “*metabolic patterns and checkpoints*” revealed by the PET-driven decision support are predictive in the treatment of cancer cachexia and thus improve the quality of the patient’s life and survival. Sociological exploration of the role of clinical decision support algorithms in clinical practice aims to enhance their comprehensibility and hopefully open new avenues to controlling cachexia.

The LuCaPET consortium, 09 FEB 2024.

## Data Availability

Not applicable.
